# Self-motivated medical care-seeking behaviors and disease progression in a community-based cohort of chronic hepatitis B virus-infected patients in China

**DOI:** 10.1186/s12889-019-7061-1

**Published:** 2019-07-08

**Authors:** Hui Zheng, Guomin Zhang, Fuzhen Wang, Zundong Yin, Ning Miao, Xiaojin Sun, Xiaofeng Liang, Fuqiang Cui

**Affiliations:** 1 National Immunization Program, Chinese Center for Disease Control and Prevention, Beijing, China; 2 Department of Laboratorial Science and Technology & Vaccine Research Center, School of Public Health, Peking University, Beijing, China

**Keywords:** Hepatitis B, Chronic infection, Medical care behaviors

## Abstract

**Background:**

To determine the treatment behaviors among a community-based cohort of chronic hepatitis B virus (HBV)-infected persons and to examine the disease progression among non-antiviral-treated HBV-infected cases after 5 years of follow-up.

**Methods:**

We conducted a community-based prospective study on people with chronic HBV infection in mainland China from 2009 to 2014. In 2009, we recruited participants who were identified as HBV infected in 2006 in a national sero-survey. A face-to-face follow-up investigation was completed in 2014, and the personal information, the clinical diagnosis provided at the last hospital visit, the HBV antiviral treatment history, and the insurance type was collected for each patient for analysis. Multivariable logistic regression was used to identify factors that are associated with active medical care- seeking and antiviral treatments.

**Results:**

Among the 2422 chronic HBV-infected patients recruited in 2009, 1784 (73.7%) were followed-up to 2014, and 638 (35.8%) had sought medical care in hospitals; among them, 140 (21.9%) received antiviral treatments. The lowest medical care-seeking rate (26%) was in participants over 50-year old. We determined that the frequency of medical care-seeking was higher among those participants living in urban areas (aRR = 1.3, 95% *CI*:1.0–1.6), those in 0–19-year old (aRR = 1.5, 95% *CI*:1.1–2.1), 20–39-year old (aRR = 2.2, 95% *CI*:1.7–3.0) and 40–49-year old (aRR = 1.5, 95% *CI*:1.1–2.0), and persons with insurance of the type Urban residents’ basic medical insurance (URBMI) or Commercial health insurance (CHI) (aRR = 2.5, 95% *CI*:1.7–3.6) and New Rural Cooperative Medical System (NRCMS) (aRR = 1.9, 95% *CI*:1.4–2.6). Patients were more likely to receive antiviral treatment if they were 20–39-year old (aRR = 0.4, 95% *CI*:0.3–0.7), had insurance of the type URBMI or CHI (aRR = 2.6, 95% *CI*:1.1–6.3) or NRCMS (aRR = 3.0, 95% *CI*:1.3–6.9) and were treated at prefecture and above-level hospitals (aRR = 2.0, 95% *CI*:1.4–3.0). Among non-antiviral-treated HBV-infected cases, we found the annual rates for HBsAg sero-clearance, progress to cirrhosis and HCC were 1.0, 0.6 and 0.2%, respectively.

**Conclusion:**

The rates of medical care-seeking and antiviral treatment were low among community-based chronic HBV-infected persons, thus we recommend improving the insurance policies for HBV-infected persons to increase the antiviral treatment rate, and conducting extensive education to promote HBV-infected patients actively seeking medical care from hospitals.

## Background

Chronic viral hepatitis is a major threat to public health worldwide, with nearly 257-million people living with chronic hepatitis B virus (HBV) infection [1]. Carriers of HBV are at increased risk for hepatocellular carcinoma (HCC) and end-stage liver diseases [2, 3]. Approximately 600-thousand to 1-million people die every year from these serious consequences of HBV infection [2]. The WHO advocates the adoption of an expanded and accelerated action to reduce the HBV burden and to eliminate HBV as a great public threat by 2030 [4]. Growing evidence has shown that antiviral therapy is effective and can reduce the risk of HCC development in chronic hepatitis B (CHB); hence, early diagnosis of and antiviral treatment for CHB are crucial for reducing morbidity and mortality [5–8].

In China, despite the success of neonatal vaccination since 1992, which has achieved a 97% reduction in the HBsAg seroprevalence from 9.7% in 1992 to 0.32% in 2014 among children under 5 years old [9, 10], there are still great challenges; nearly 90-million people living with HBV infection [11]. Recent surveillance data show HCC to be ranked as the third leading cause of cancer-related deaths in China, and 80–85% of those deaths are caused by HBV infection [12–14]. This heavy HBV-related disease burden is a cause for great concern, and it is expected that the death rate from HBV infection will continue to increase if no actions are taken. Therefore, there is a need for a comprehensive strategy for the control and prevention of hepatitis B that goes beyond vaccination and includes the early detection and treatment of chronic hepatitis B as key elements.

Numerous studies from China have demonstrated a successful vaccination history in reducing the HBsAg prevalence from 1979 to 2014 [10, 11, 15]. There are fewer available data regarding community-based chronic HBV-infected persons who need medical care and treatment, estimating the resources needed for rendering medical service to them. Furthermore, most previous studies on the medical behaviors of HBV-infected persons or the antiviral treatment analysis thereof were focused on the hospital-based populations [16–19]; very few related studies have been conducted on community-based populations.

We designed and conducted a prospective study on community-basedHBV-infected persons to analyze their medical care-seeking behaviors and their antiviral treatment situation, as well as the related factors; for HBV-infected cases without anti-viral treatment, we observed the disease progression after a 5-year follow-up.

## Methods

### Cohort establishment and study design

We performed a community-based prospective follow-up study on a chronic HBV-infected cohort to observe their self-motivated medical care-seeking behaviors. In 2009, we revisited the chronic HBV-infected persons who were shown to be HBsAg-positive in a 2006 national sero-survey that employed a multistage random sampling method [9], and then we recruited target subjects with the following inclusion criteria: 1) reliable HBsAg-positive test results; 2) local resident for at least 6 months; 3) no other chronic liver diseases, such as coinfection with hepatitis C virus (HCV) or hepatitis D virus **(**HDV), alcoholic or nonalcoholic fatty liver disease or steatohepatitis; 4) willingness to participate in the investigation.

### Diagnostic criteria for HBV infection

Each participant was clinically examined for any signs and symptoms of liver disease, after which routine blood tests, including alanine transaminase (ALT), and an abdominal ultrasound were performed. According to the national guidelines [20, 21], hepatitis B patients should be classified as follows: (1) carrier: HBsAg-positive cases with no symptoms of liver diseases (e.g., nausea, vomiting, diarrhea, anorexia, abdominal pain, and jaundice) and normal ALT levels (≤40 IU/mL); (2) CHB: HBsAg-positive cases with abnormal ALT levels (> 80 IU/mL) and at least one of the following: HBV infection ≥6 months prior, chronic inflammatory changes reported on abdominal ultrasound, or anti-HBc IgM negative; (3) liver cirrhosis (LC): HBsAg-positive cases with liver cirrhosis as reported via abdominal ultrasound, computerized tomography (CT) or Magnetic Resonance Imaging (MRI); (4) HCC: cases with liver lesion(s) suggestive of hepatocellular carcinoma reported via abdominal ultrasound, CT or MRI together with alpha fetoprotein (AFP) > 400 μg/mL; and (5) HBsAg sero-clearance: cases with HBsAg changing from positive to negative, positive or negative for anti-HBs.

### Follow-up investigation and data collection

We conducted face-to-face follow-up appointments in 2014, and data were collected by trained staff in 2014. We collected the primary information on medical care behaviors and disease progression and reviewed the case information according to the above mentioned criteria. The data included personal information, the clinical diagnosis at the last visit, which was provided by the hospital, the antiviral treatment history (including Tenofovir, Entecavir, Adefovir, Lamivudine, and PEG-IFN), and the insurance status. The patients were classified as having self-motivated medical care behavior for HBV-related diseases by providing related evidence during the study period, such as hospital diagnostic records, prescriptions or hospital testing results.

### Quality control

In 2009, for each participant, the clinical diagnosis was reconfirmed by national hepatitis clinical experts. Regular communications were conducted to reduce the loss to follow-up. In 2014, we used a unified questionnaire to investigate the medical care-seeking behaviors; the questionnaire was administered by trained staff, and the subsequent clinical diagnosis and the treatment history were demonstrated by obtaining copies of the hospitals’ documents.

### Statistical analysis

All the data were double-entered using EPI Data 3.1 (EPI Data Association, Odense, Denmark) and checked for consistency. We used SPSS software (version 23, IBM, United States) and SAS software (version 9.4, SAS Institute, Inc., Cary, NC, USA) for the statistical analyses. Descriptive statistical methods were used to analyze the demographic characteristics and the proportion of disease status among HBV-infected persons. The chi-square test and multivariate regression analyses [presented as the relative risk (RR) with 95% confidence intervals (*CI*)] were used to compare the risk factors related to the active medical care-seeking behaviors and antiviral treatments of HBV-infected persons. The estimation of the rates of disease progression and HBsAg sero-clearance were calculated by a person-year using the life table method. Statistical significance was considered for those results with a two-tailed *p* < 0.05.

### Ethical issues

This study was approved by the China CDC Ethical Review Committee, and the study was conducted following the ethics regulations. All participants were informed of the study purpose and their right to keep information confidential.

## Results

**Fig. 1:**
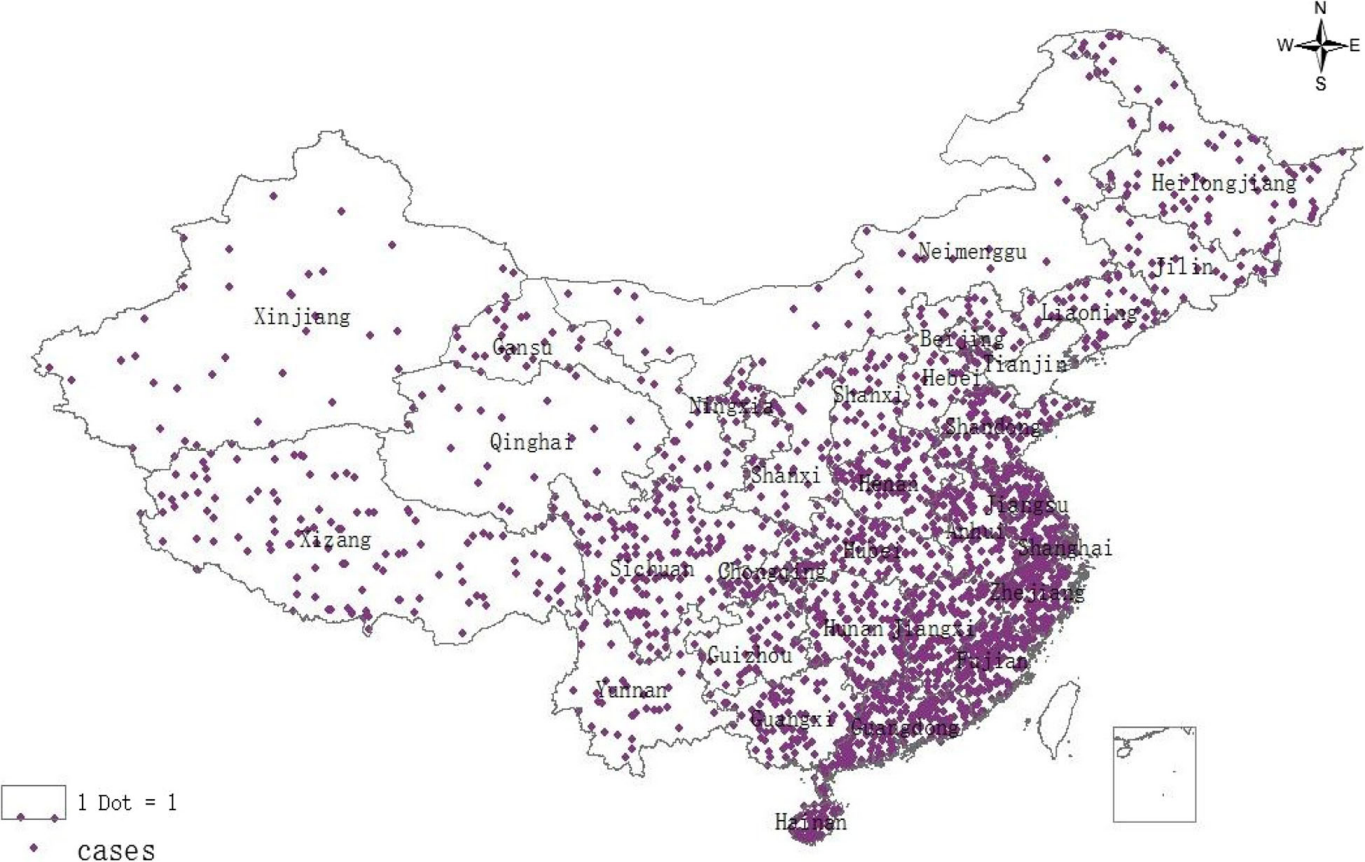
Sample distribution of chronic HBV-infected cohort in China, 2009–2014

**Fig. 2:**
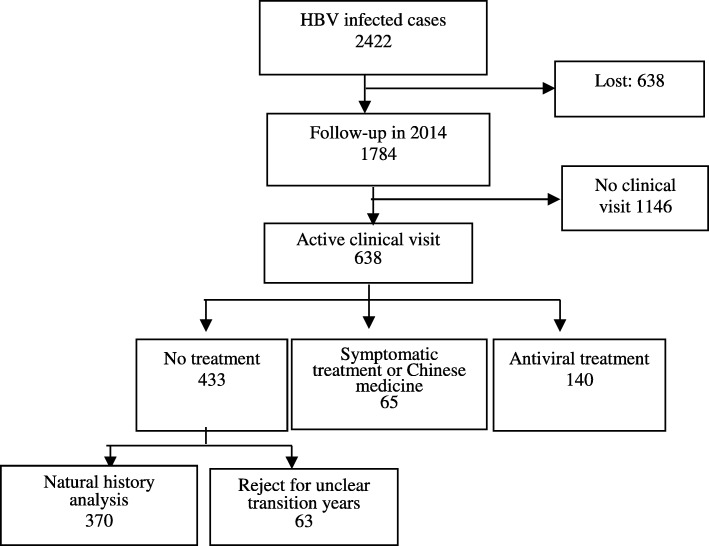
Chronic HBV-infected people enrolled and cohort followed-up, China, 2009–2014.

A total of 2422 hepatitis B virus-infected patients were enrolled from 31 provinces in China in 2009 (Fig. 1); the demographic characteristics of the cohort are shown in Table 1. Among the participants, 1784 (74%) were followed up to 2014. No significant differences were detected between followed-up persons and lost to follow-up (LTFU) persons in terms of gender (*P* = 0.27) and area of residence (rural or urban) (*P* = 0.16) (Fig. 2), Table 1).

**Table 1:** The demographic characteristics of follow-up cases and lost to follow-up cases in the HBV-infected cohort, China, 2009

	Followed-up [n, (%)]				Lost to follow-up [n, (%)]				Total [n, (%)]			
	Carrier	CHB^a^	LC^b^	HCC^c^	Carrier	CHB	LC	HCC	Carrier	CHB	LC	HCC
Gender												
Male	633	318	15	1	184	139	5	1	817	457	20	2
	(53.6)	(55.1)	(62.5)	(50.0)	(49.9)	(53.1)	(100)	(50.0)	(52.7)	(54.5)	(69.0)	(50.0)
Female	548	259	9	1	185	123	0	1	733	382	9	2
	(46.4)	(44.9)	(37.5)	(50.0)	(50.1)	(46.9)	(0.0)	(50.0)	(47.3)	(45.5)	(31.0)	(50.0)
Age group												
0–19	213	51	2	0	101	44	0	0	314	95	2	0
	(18.0)	(8.8)	(8.3)	(0.0)	(27.4)	(16.8)	(0.0)	(0.0)	(20.3)	(11.3)	(6.9)	(0.0)
20–39	400	184	4	0	121	84	0	0	521	268	4	0
	(33.9)	(31.9)	(16.7)	(0.0)	(32.8)	(32.1)	(0.0)	(0.0)	(33.6)	(31.9)	(13.8)	(0.0)
40–49	322	179	8	1	64	67	2	0	386	246	10	1
	(27.3)	(31.0)	(33.3)	(50.0)	(17.3)	(25.6)	(40.0)	(0.0)	(24.9)	(29.3)	(34.5)	(25.0)
50–62	246	163	10	1	83	67	3	2	329	230	13	3
	(20.8)	(28.2)	(41.7)	(50.0)	(22.5)	(25.6)	(60.0)	(100.0)	(21.2)	(27.4)	(44.8)	(75.0)
Region												
East	569	235	14	2	90	53	2	1	659	288	16	3
	(48.2)	(40.7)	(58.3)	(100)	(22.4)	(20.2)	(40.0)	(50.0)	(42.5)	(34.3)	(55.2)	(75.0)
Central	321	225	8	0	144	84	3	1	465	309	11	1
	(27.2)	(39.0)	(33.3)	(0.0)	(39.0)	(32.1)	(60.0)	(50.0)	(30.0)	(36.8)	(37.9)	(25.0)
West	291	117	2	0	135	125	0	0	426	242	2	0
	(24.6)	(20.3)	(8.3)	(0.0)	(36.6)	(47.7)	(0.0)	(0.0)	(27.5)	(28.8)	(6.9)	(0.0)
Rural/Urban												
Urban	430	249	9	0	154	110	3	0	584	359	12	0
	(36.4)	(43.2)	(37.5)	(0.0)	(41.7)	(42.0)	(60.0)	(0.0)	(37.7)	(42.8)	(41.4)	(0.0)
Rural	751	328	15	2	215	152	2	2	966	480	17	4
	(63.6)	(56.8)	(62.5)	(100)	(58.3)	(58.0)	(40.0)	(100)	(62.3)	(57.2)	(58.6)	(100.0)
**Total**	1181	577	24	2	369	262	5	2	1550	839	29	4
	(100)	(100)	(100)	(100)	(100)	(100)	(100)	(100)	(100)	(100)	(100)	(100)

^a^ *CHB* chronic hepatitis B

^b^ *LC* liver cirrhosis

^c^ *HCC* hepatocellular carcinoma

In 2009, we confirmed that 1550 (64%) of the participants were HBV carriers, 839 (34.6%) had CHB, 29 (1.2%) had LC and 4 (0.2%) had HCC. Significant differences in HBV infection status existed among age-groups (*P* < 0.001) and regions (*P* < 0.001). All the HCC cases and nearly two-thirds of the LC cases occurred in rural areas (Table 1).

In 2014, 64.2% (1146/1784) of the follow-ups had not visited a clinic. Among the 638 participants who had actively sought medical care, 433 (67.9%) received a medical examination but no medication, 65 (10.2%) took Chinese traditional herbal medicine, and there were 140 (21.9%) cases who received antiviral treatment (Table 2). The participants’ self-reported reasons for not visiting a hospital included the following: 1) 92.8% (1063/1164) claimed not to experience any liver disease symptoms; 2) 10.0% (115/1164) indicated that the hospital cost was high; and 3) 6.3% (76/1164) were concerned about their privacy.

**Table 2:** The active medical care-seeking behaviors and treatment situations among chronic HBV-infected persons, China, 2009–2014

Medical activities	Disease status	Sample Size	Treatment category					
			No treatment		Symptomatic treatment or Chinese medicine		Antiviral treatment	
			n	%	n	%	n	%
No clinical visit	Carriers	773	773	100	–	–	–	–
	CHB^a^	361	361	100	–	–	–	–
	LC^b^	12	12	100	–	–	–	–
	Sub-total	1146	1146	100				
Active clinical visit	Carriers	408	289	70.8	30	7.4	89	21.8
	CHB	216	137	63.4	33	15.3	46	21.3
	LC	12	7	58.3	0	0.0	5	41.7
	HCC^c^	2	0	0.0	2	100.0	0	0.0
	Sub-total	638	433	67.9	65	10.2	140	21.9
Total	Carriers	1181	1062	89.9	30	2.5	89	7.5
	CHB	577	498	86.3	33	5.7	46	8.0
	LC	24	19	79.2	0	0.0	5	20.8
	HCC	2	0	0.0	2	100.0	0	0.0
	Sub-total	1784	1579	88.5	65	3.6	140	7.8

^a^ *CHB* chronic hepatitis B

^b^ *LC* liver cirrhosis

^c^ *HCC* hepatocellular carcinoma

In our bivariate analysis, we detected significant differences in both the active medical care-seeking behaviors and antiviral treatments of chronic HBV-infected persons according to age-group and insurance type. In addition, significant differences were detected the active medical care-seeking behaviors of chronic HBV-infected persons according to region and living in a rural vs urban area (all *p*-values < 0.05) (Table 3). In the multivariable analysis, participants were more likely to actively seek medical care if they were urban (aRR = 1.3, 95% *CI*:1.0–1.6), 0–19 years old (aRR = 1.5, 95% *CI*:1.1–2.1), 20–39 years old (aRR = 2.2, 95% *CI*:1.7–3.0) 40–49 years old (aRR = 1.5, 95% *CI*:1.1–2.0), with insurance of Urban residents’ basic medical insurance (URBMI) or Commercial health insurance (CHI) (aRR = 2.5, 95% *CI*:1.7–3.6) and New Rural Cooperative Medical System (NRCMS) (aRR = 1.9, 95% *CI*:1.4–2.6). In addition, patients were more likely to receive antiviral treatments if they were 20–39 years old (aRR = 0.4, 95% *CI*:0.3–0.7), with insurance of URBMI or CHI (aRR = 2.6, 95% *CI*:1.1–6.3) and NRCMS (aRR = 3.0, 95% *CI*:1.3–6.9) and if they were treated at a prefecture and above-level hospital (aRR = 2.0, 95% *CI*:1.4–3.0) (Table 3).

**Table 3:** The risk factors associated with the active medical care-seeking behaviors and antiviral treatments in chronic HBV-infected persons, China, 2009–2014

	Sample size	Active clinical visit				Sample size	Antiviral treatment			
		Crude analysis		Multivariable analysis			Crude analysis		Multivariable analysis	
		n	*P-*Value	aRR (95% *CI*)	*P*-Value		n	*P-*Value	aRR (95% *CI*) *CI*)	*P*- Value
Region										
East	820	266	0.03	0.78 (0.61–1.01)	0.06	266	56	0.12		
Central	554	216		1.02 (0.87–1.34)	0.88	216	57		–	–
West	410	156		1		156	28			
Rural/Urban										
Urban	688	275	0.004	1.3 (1.0–1.6)	0.04	275	62	0.77	–	–
Rural	1096	363		1		363	78			
Gender										
Male	967	341	0.66	–	–	341	73	0.77	–	–
Female	817	297		–	–	297	67			
Beginning age-group										
0–19	266	90	< 0.001	1.5 (1.1–2.1)	0.017	90	20	0.004	0.7 (0.4–1.3)	0.20
20–39	588	261		2.2 (1.7–3.0)	< 0.001	261	40		0.4 (0.3–0.7)	0.001
40–49	510	178		1.5 (1.1–2.0)	0.007	178	47		0.8 (0.5–1.4)	0.44
50–62	420	109		1		109	33		1	
Insurance type										
URBMI^a^ or CHI^b^	320	146	< 0.001	2.5 (1.7–3.6)	< 0.001	146	34	0.04	2.6 (1.1–6.3)	0.04
NRCMS^c^	1175	424		1.9 (1.4–2.6)	< 0.001	424	99		3.0 (1.3–6.9)	0.01
None or unclear	289	68		1		68	7		1	
Hospital level										
Prefecture and above	–	–	–	–	–	294	82	0.003	2.0 (1.4–3.0)	< 0.001
County and below	–	–				344	58		1	
Total	1784	638	–			638	140	–		

^a^*URBMI* Urban residents’ basic medical insurance,^b^ *CHI* Commercial health insurance,^c^ *NRCMS* New Rural Cooperative Medical System

We analyzed the natural disease progression among 370 chronic hepatitis B patients who did not accept any antiviral therapy. During 1212 person-years of follow-up, the annual rates for HBsAg sero-clearance, progress to cirrhosis and HCC were 1.0, 0.6 and 0.2%, respectively (Table 4).

**Table 4:** The disease progress at the 5-year follow-up, China, 2009–2014

Initial diagnosis	Person-years	Follow-up diagnosis									
		HBsAg sero-clearance		Carriers		CHB^a^		LC^b^		HCC^C^	
		n	Annual rate (%)	n	Annual rate (%)	n	Annual rate (%)	n	Annual rate (%)	n	Annual rate (%)
Carriers	821	11	1.3	185	22.5	56	6.8	0	–	0	–
CHB	391	1	0.3	0	–	108	–	2	0.5	0	–
LC	29	0	–	0	–	0	–	5	17.2	2	6.9
Total	1212	12	1.0	185	15.3	164	4.6	7	0.6	2	0.2

^a^ *CHB* chronic hepatitis B

^b^ *LC* liver cirrhosis

^c^ *HCC* hepatocellular carcinoma

Among the carriers, we determined that 11 persons had HBsAg sero-clearance, and 56 persons progressed to CHB during 821 person-years of follow-up, making the annual rate of sero-clearance and CHB 1.3 and 6.8%, respectively. Among the CHB participants, only one person had HBsAg sero-clearance, and two persons experienced cirrhosis after 391 person-years of follow-up, yielding annual rates of sero-clearance and cirrhosis of 0.3 and 0.5%, respectively. During 29 person-years of follow-up for participants with cirrhosis, two persons developed HCC, yielding an annual probability of cirrhosis to HCC of 6.9% (Table 4).

## Discussion

Studies have suggested that among the 90-million HBV-infected people in China, 28-million are chronic active hepatitis B patients with symptoms who need to be treated, based on the model [11, 22–24]. In contrast, studies exploring the different disease status of community-based HBV infected persons are limited, although such studies are the basis for the scientific estimation of the HBV burden and health resource allocation.

In this study, we identified that among HBV-infected cases, nearly 64% were carriers and 35% had CHB, which was a finding that was very similar to previous estimations that CHB would account for 1/3 of HBV-infected people [25, 26]. In China, 80–90% of the chronic HBV infections were acquired through mother-to-child transmission (MTCT) in the prevaccine era, and most of these cases were in the immune tolerance stage with normal ALT and without obvious inflammation of the liver [25]. Our results demonstrate that with increasing age, the proportions of CHB (from 23 to 40%), LC (from 0.5 to 2.3%) and HCC (from 0 to 0.5%) increased, whereas the carrier proportion decreases from 76 to 57%. In contrast, Yang SG [27] reported that the proportion of CHB among HBsAg-positive persons was only 3.98% in a community-based study in Zhejiang province. The difference may be due to differences in the diagnostic criteria, as 95% of HBsAg-positive persons were classified as HBV carriers in that study, which is much higher than is generally acknowledged. Among cases not receiving any treatment, the annual person-year HBsAg sero-clearance rate was nearly 1.0%, and most of the sero-clearance occurred in asymptomatic carriers, which is similar to results from previous studies [28]. Without antiviral intervention, 0.5% of the CHB patients would develop cirrhosis annually; however, the progress from cirrhosis to HCC was nearly 7%. To interrupt the progress from active chronic hepatitis B to cirrhosis and in turn slow the development from cirrhosis to HCC, which is the primary target of HBV treatment, regular hospital examinations and antiviral treatment at the appropriate starting point were shown to be important.

We determined that the self-motivated clinic visitation rate is low; it was only 35%, even among those who are aware they were infected with HBV. Regular medical examinations can monitor the disease, and effective measures can be taken to slow the disease progression when necessary [29]. Studies have demonstrated that people’s misunderstanding of the goals and the effectiveness of treatments greatly influence the medical behaviors of HBV carriers [23, 30]. In our study, we determined that nearly 93% of persons who exhibited no self-motivated medical behaviors misunderstood their symptoms and did not believe that they needed to go to the hospital, which suggests that the health education targeted at such individuals should be strengthened to promote their knowledge of the importance of regular medical examinations. In addition, 6% of the LTFUs reported fear of their HBV infection being known by others. Such data reflects that discrimination related to hepatitis B exists in China [31–33] and affects people’s medical behavior to some extent.

The rate of antiviral treatment for hepatitis B in China was shown to be low. According to our study, the rate of antiviral treatment among community-based HBV-infected persons was only approximately 8%, which is much lower than the hospital-based estimation of 12–19% [34]. Based on the modeling estimation by the WHO, HBV-related mortality in China will rise continuously in the next 30–40 years, given the present 12.5% antiviral treatment rate, unless we increase this rate to 50% of CHBs, which could greatly reduce the rate of HBV-related death for LC and HCC in 5 years [35]. Meanwhile, over-treatment also occurs. We determined that 7.5% of HBV carriers had taken antiviral medicine even when such treatment was unwarranted [29]. Inappropriate antiviral treatment does not have a good effect; instead, it poses an economic burden for families and wastes medical resources.

Compared to young people aged 20–39-yrs, the self-motivated medical visitation rate was lower among those over 40-yrs old, who should be treated as priority because they are subject to higher risks of developing LC and HCC compared with young persons without medical intervention [29]. Previous studies have demonstrated that the health care knowledge on hepatitis B is poor among elderly people, and their limited or incorrect knowledge of hepatitis B results in negative medical behaviors [36, 37]. However, we also determined that although the proportion of young people seeking medical treatment was higher, the proportion of young people who received antiviral treatment was very low, which is partly because some young HBV-infected cases do not meet antiviral indications. However, the lack of antiviral treatment was largely because young patients, especially those under the age of 30, felt asymptomatic and did not take medication. In addition, we determined that the rural population had a lower rate of active medical care- seeking behavior compared with those living in urban areas, which may explain why we showed rural persons to be more vulnerable, having serious consequences of 60% LC and 100% HCC rates diagnosed in rural areas at the beginning of this study. These results suggest that we should pay more attention to the education and management of HBV-infected people aged over 40 years, especially those in the rural areas.

We also determined that the type of insurance affects people’s medical care-seeking behaviors. Those without insurance exhibited a lower active clinic visitation rate and antiviral treatment rate compared with those who were insured. This phenomenon reflects the huge economic burden of hepatitis B treatment. In China, nearly 70% of CHB patients’ per capita annual income was lower than $1500, and for 90% of them the cost of hepatitis B testing and treatment was unaffordable. For example, the monthly cost of the antiviral drug tenofovir (TDF), which is used to treat hepatitis B, was reduced greatly from $220 to $70 in 2016 [38], but this cost is still 4 times the price for the same dosage for HIV antiviral treatment ($17/month). The policies regarding reimbursement for hepatitis B antiviral drugs varied across the different insurance types and from province to province, ranging from nearly $300 to $3000 for URBMI (which primarily covered the urban population normally with higher reimbursement than NRCMS) or CHI (the applicant purchases commercial insurance from an insurance company at his own expense according to his personal economic ability and willingness). For the people covered by NRCMS, who were mostly in rural areas, the reimbursement rate was the lowest among these three types of insurance. This phenomenon may also be one of the reasons why the treatment rate is lower among patients who went to the prefecture or higher level hospitals compared with those who went to county or lower level hospitals, as we demonstrated in this study. We expect that if the antiviral treatment price declines, treatment coverage will rise.

Several limitations exist in this study. Firstly, the sample sizes are small, and the follow-up time is 5 years, both of which may affect the results. Secondly, we determined that 26% of the cohort dropped out in 2014, which may overestimate or underestimate the rate of self-motivated medical treatment seeking. Thirdly, there may be some recall bias in our study.

## Conclusion

We identified that the proportions of the carriers and CHB among chronic HBV-infected persons in China were 64 and 35%, respectively. The rate of self-motivated seeking of antiviral treatment was lower, with only one-third of these community-based participants making active clinic visits and 8% taking antiviral therapy. The risk factors for failing to seek medical treatment included age greater than 40, low insurance reimbursement and residence in a rural area. Among the persons without medical intervention, only 1.0% exhibited HBsAg sero-clearance annually, and the progression from cirrhosis to HCC was rapid at an annual rate of 7%.

## Data Availability

Because of data protection, the data generated and analyzed during the current study are not published, but the appropriate authors may obtain access to and/or analyze the data from the current study upon reasonable request.

## Abbreviations

95% CI: 95% Confidence interval
AFP: Alpha fetoprotein
ALT: Alanine transaminase
CHB: Chronic hepatitis B
CHI: Commercial health insurance
CT: Computerized tomography
HBV: Hepatitis B virus
HCC: Hepatocellular carcinoma
HCV: Hepatitis C virus
HDV: Hepatitis D virus
LC: Liver cirrhosis
LTFU: Lost to follow-up
MRI: Magnetic Resonance Imaging
NRCMS: New Rural Cooperative Medical System
RR: Relative risk
TDF: Tenofovir
URBMI: Urban residents’ basic medical insurance
